# Soft X-Ray Microscopy Radiation Damage On Fixed Cells Investigated With Synchrotron Radiation FTIR Microscopy

**DOI:** 10.1038/srep10250

**Published:** 2015-05-14

**Authors:** A. Gianoncelli, L. Vaccari, G. Kourousias, D. Cassese, D. E. Bedolla, S. Kenig, P. Storici, M. Lazzarino, M. Kiskinova

**Affiliations:** 1 Elettra – Sincrotrone Trieste, 34149, Basovizza, Trieste, Italy; 2 IOM-CNR Laboratorio TASC, 34149, Basovizza, Trieste, Italy; 3 Physics Department, University of Trieste, 34127 Trieste, Italy

## Abstract

Radiation damage of biological samples remains a limiting factor in high resolution X-ray microscopy (XRM). Several studies have attempted to evaluate the extent and the effects of radiation damage, proposing strategies to minimise or prevent it. The present work aims to assess the impact of soft X-rays on formalin fixed cells on a systematic manner. The novelty of this approach resides on investigating the radiation damage not only with XRM, as often reported in relevant literature on the topic, but by coupling it with two additional independent non-destructive microscopy methods: Atomic Force Microscopy (AFM) and FTIR Microscopy (FTIRM). Human Embryonic Kidney 293 cells were exposed to different radiation doses at 1 keV. In order to reveal possible morphological and biochemical changes, the irradiated cells were systematically analysed with AFM and FTIRM before and after. Results reveal that while cell morphology is not substantially affected, cellular biochemical profile changes significantly and progressively when increasing dose, resulting in a severe breakdown of the covalent bonding network. This information impacts most soft XRM studies on fixed cells and adds an in-depth understanding of the radiation damage for developing better prevention strategies.

X-ray microscopy has become a powerful tool for imaging complex biological systems at cellular and sub-cellular scale since it combines submicrometer spatial resolution, penetration power and chemical sensitivity. Scanning X-ray microscopy at synchrotron facilities can deploy different imaging modes coupled with spectroscopic capabilities (X-ray Fluorescence and X-ray Absorption Near Edge Structure), providing simultaneous morphological and chemical information on the specimen under analysis^2^.

However, the extent to which the lateral resolution can be pushed without unacceptable sample degradation is still an open question. Due to the intrinsic sensitivity of biological matter, finding the best compromise between required dose for imaging and maximum tolerable dose for bio-samples represents the keystone for the applicability/reliability of any technique to life-sciences^3^. In fact, radiation damage remains a limiting factor for imaging samples at high resolution^3^, especially when operating in the soft X-ray energy regime. Soft X-rays cause more damage than hard X-rays due to the high absorption cross sections of C, N and O K-shells, the main organic matter constituents. It is well recognized that the extent of the radiation damage depends on different factors, such as X-ray wavelength, exposure dose, sample preparation and environment. However, a comprehensive, complete and thorough understanding of the issues involved in the process is still lacking. Since realistic dosages for high resolution X-ray imaging can be of the order of 10^6^ Gy^4^, imaging of living cells is only possible with impractical sub-nanosecond exposures, i.e. prior to the manifestation of radiation-induced structural disintegrity^5^. It has been reported that the main radiation damage for chemically fixed samples is mass thickness loss and sample shrinkage^6^, whereas for fully hydrated samples such doses can damage living bacteria^7^ and cause apoptosis, chromosomal aberration, DNA mutations and loss in the reproduction ability of living cells^6^. Williams *et al.*^6^ investigated radiation damage on freeze-dried and fixed (glutaraldehyde or formaldehyde or osmium tetroxide) hydrated chromosomes in the water window highlighting once more that for a given dose dried specimens undergo less radiation damage than wet ones. Fujisaki and co-authors^8^ investigated the effect of radiation dose on yeast cells showing that they lose their dye exclusion ability above specific doses. The same authors noticed that myofibrils lose contractility according to the delivered dose, as previously observed by Bennett and co-authors^9^. Despite these interesting findings, a systematic study on the biochemical response of these systems to irradiation is still lacking.

For X-ray microscopy, the estimation of tolerated doses and structural changes becomes especially important when performing quantitative elemental mapping. The literature reports that for fully hydrated ascidian blood cells, analysed at room temperature with hard X-rays (5 KeV), the tolerated doses are too low to allow detecting ppm concentrations of trace elements^0^. In fact, without cryo-preservation only faster and more sensitive detectors can allow low detection limit elemental mapping with a suitable dose that would not cause any significant mass thickness loss^0^. Indeed, mass thickness loss can sensibly be reduced by using cryo-methods^1^, which preserve the cellular morphology, preventing the diffusion of free radicals created as result of X-ray damage^2^. However, as demonstrated by Beetz and Jacobsen^1^, the C = O bond breaking rate in Poly(methyl methacrylate), PMMA, is the same for cryo- and room temperature experiments, while the oxygen mass loss can be reduced at liquid nitrogen temperatures. Wang and co-authors also assessed the radiation damage induced by STXM and X-PEEM in PMMA, Polysterene and Plasminogen-free human plasma fibrinogen, by comparing C 1s, N 1s and O 1s spectra in different (exposed and not exposed) locations^3^. They show that PMMA critical dose is 4 times smaller than for Plasminogen-free human plasma fibrinogen and around 18 times than for polystyrene. After spectra evaluation, they propose different reactions mechanisms for the three analysed samples, demonstrating how important is the specimen matrix composition. The set of radiation damage reactions appears to be quite complicated, but in line previous studies performed on individual amino acids^4^ where the radiation damage can induce dehydration, decarboxylation, decarbonylation, deamination and desulfurisation together with desorption of H_2_, H_2_O, CO_2_, CO, NH_3_ and H_2_S^5^. Moreover, they identified formation of C-C and C-N double and triple bonds following soft X-ray exposure. This could be assessed thanks to the combination of XPS, mass spectrometry and NEFAXS studies. Zubavichus and co-authors^5^ also found that various amino acids present different stability to soft X-ray radiation. This shows once more that despite these interesting and useful outcomes, it is difficult to extend these results to organic cellular matter, hydrated or dried. Moreover, as mentioned above, it is expected that the effect of radiation dose will be different on fixed and hydrated cells, being more accentuated on the latter. Another study, based on XANES experiments at room temperature and at –100 °C, shows that mass thickness loss and photoreduction processes can be slowed down but not eliminated by reducing the working temperature^6^.

STXM and Carbon XANES measurements on plant cell walls showed that polysaccharides are susceptible to soft X-ray radiation^7^. In particular, Cody and co-authors^7^ identified mass loss compatible with de-acetylation as effect of soft X-ray radiation damage on cellulose acetate. In addition, results on oak cells revealed that cellulose and hemicellulose are less sensitive to radiation damage than cellulose acetate, once again demonstrating the complexity of the radiation damage issue and its dependence on the matrix composition. A similar study on the organic matter of clay-rich mineral by C 1s XANES shows formation of C = C caused by crosslinking via polymerisation and mass loss (20–30% oxygen and 13–18% carbon) from bond breaking in organic macromolecules^8^.

Nevertheless, all results reported in literature are based on processing and evaluating the absorption and/or phase contrast images, therefore, they are obtained using the very same technique for inducing and estimating the radiation damage effect. In this paper, we adopt a novel approach based on the use of three microscopy techniques, so that the effects of Scanning Transmission X-ray Microscopy (STXM) and low energy X-ray Fluorescence (LEXRF) are evaluated by two other non-damaging methods: Atomic Force Microscopy (AFM) and Fourier Transform Infrared Microscopy (FTIRM), consequently, decoupling the source of the damage from the probe of it.

AFM is a non-invasive microscopy technique that may produce a detailed 3D reconstruction of a sample at nanometric resolution. Thanks to its capability of operating both in air and in liquid, it has been largely employed to investigate the morphology of biological specimens at cellular and sub-cellular level, both from fixed^9^ and living samples, and to correlate volume variations of a physiological process^0^. Hence, it represents the ideal technique to assess the volume and thickness variation of a biological sample exposed to X-ray radiation.

The evaluation of biochemical changes was instead performed at sub-cellular lateral resolution by Synchrotron Radiation (SR) FTIRM. The advantages of FTIRM are the label-free and non-destructive characteristics, since it probes matter by using low-energy photons (0.05–0.5 eV) in the MidIR regime, exciting vibration modes of the most diagnostic cellular biomolecules, without crossing their ionization threshold. This is valid also for bright and highly collimated IR-SR light. Holman and coworkers demonstrated that the exposure of single human T1-cells to IR-SR beam up to 20 minutes does not alter cellular viability, cell-cycle progression, cellular metabolism nor it affects the cellular proliferative capacity^1^. In addition, only a minimal sample heating, around 0.5 °C, has been detected for similar exposure conditions^2^. Thus, SR-FTIRM is a methodology suitable for in-situ biochemical characterization of live cells and for monitoring their biological response without interfering with the cellular pathways.

The specific purpose of the present work is to investigate through SR-FTIRM the chemical changes induced at sub-cellular lateral resolution by soft X-rays on paraformaldehyde (PFA) fixed Human Embryonic Kidney cells (HEK293T) at exposure conditions commonly employed at the TwinMic soft X-ray microscopy beamline. Specifically, selected formalin fixed air-dried HEK293T cells were firstly characterized chemically by vibrational mapping, then dried under vacuum and lastly exposed to increasing doses of X-rays (from 2∙10^6^ to 6.2∙10^8^ Gy). At each step, investigated cells were mapped again by SR-FTIRM. Moreover, in order to get semi-quantitative information on density changes and on alteration of the biochemical cellular architecture, the cellular topography was monitored by AFM analysis at each step.

## Results and discussion

The effects of possible cellular damage induced by soft X-ray irradiation have been evaluated on three HEK293T cells (Cell1, Cell2 and Cell3 hereafter), grown on 100 nm thick silicon nitride windows, a substrate suitable for both X-ray microscopy and FTIRM, and subsequently fixed with 3.7% paraformaldehyde in Phosphate Buffer Solution (PBS) and let to air-dry overnight. Samples were analysed immediately after preparation following a systematic procedure. Initially, few PFA fixed HEK293T cells were selected under a visible light microscope and subsequently were mapped with SR-FTIRM and AFM after air drying (Step 0). At Step 1 cells were vacuum dried for 1h30 at a pressure lower than 5∙10^−5^ mbar, and then mapped again with SR-FTIRM and AFM. At Step 2, individual cells were exposed to a low X-ray dose (2∙10^6^ Gy); at Step 3, cells previously exposed were irradiated with a medium X-ray dose (2∙10^7^ Gy) in order to achieve a cumulative dose of 2.2∙10^7^ Gy; finally (Step 4) selected cells were exposed again to a higher X-ray dose (6∙10^8^ Gray), so that every cell was finally irradiated with 6.22∙10^8^ Gray cumulative dose. After each exposure step, the selected cells were mapped with SR-FTIRM and AFM. The experiment was designed in such a way as to minimize the time between one measurement and the following one, so that all steps for each cell were completed in three consecutive days. This tight scheduling allowed us to minimize potential sample aging deleterious effects, which should manifest for a much longer lag between sample fixation and sample measurement. Indeed, sample-aging adverse phenomena are expected to occur after weeks^3^ or even years of storage on slides of formalin fixed tissues^4^, especially affecting the antigenicity for proteins and DNA, while leaving substantially unaltered their content^5^ and morphology^6^.

**Figure 1:**
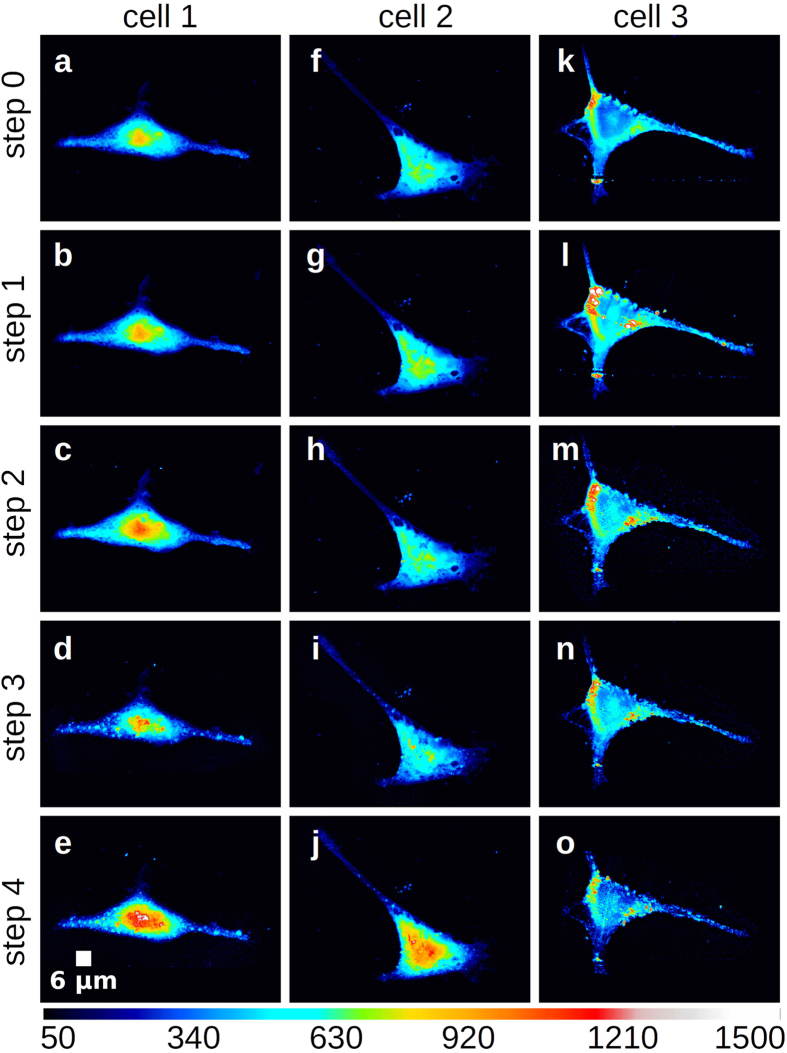
AFM images of the three analysed formalin-fixed cells. AFM images of the three analysed formalin-fixed cells (Cell1 **a**–**e**, Cell2 **f**–**j**, Cell3 **k**–**o**) during the five experimental steps: Air-dried (**a**,**f**,**k**), Vacuum-dried (**b**,**j**,l), low X-ray dose exposure compatible with low resolution STXM (**c**,**h**,**m**), medium X-ray dose exposure compatible with high-resolution STXM (**d**,**i**,**n**) and finally high X-ray dose exposure compatible with XRF (**e**,**j**,**o**).

**Figure 2:**
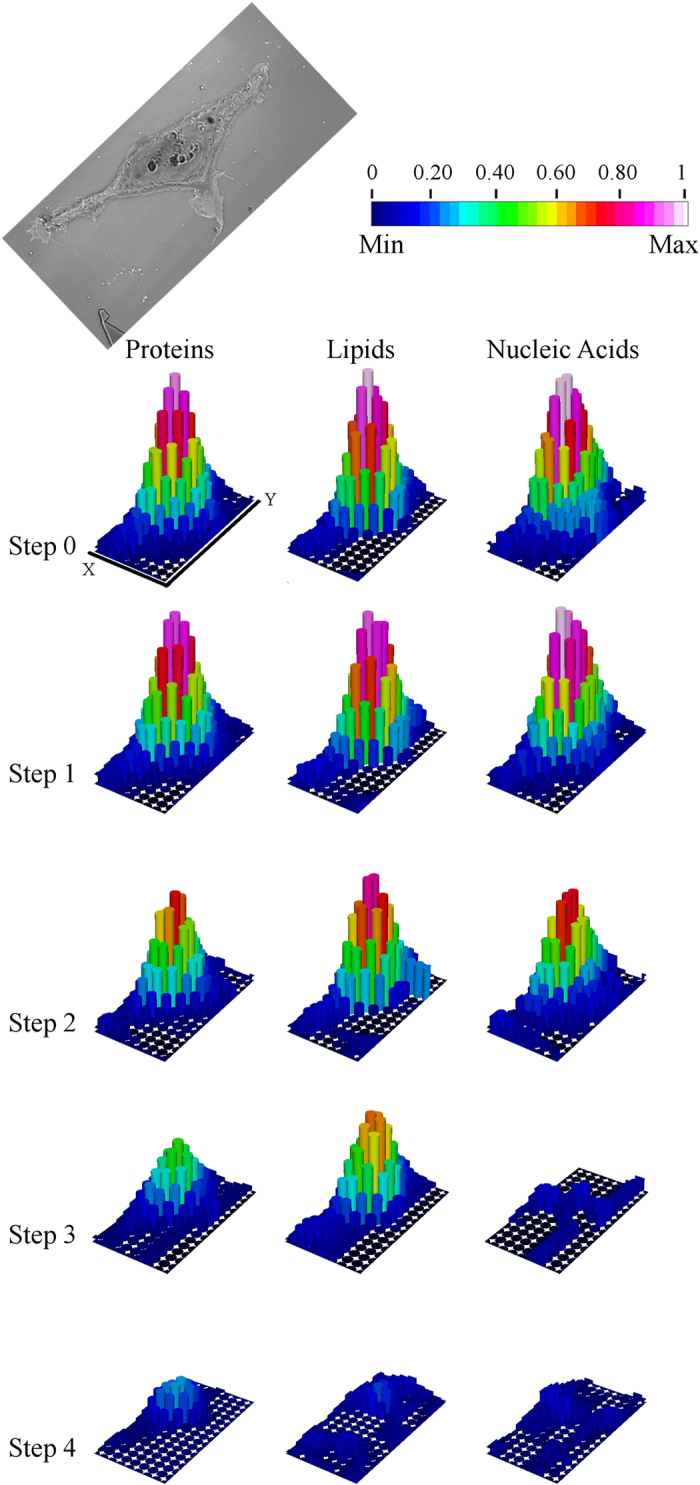
FTIR Chemical Images. Chemical images (54 × 96 μm^2^ area at 6 μm lateral resolution) of the distribution of cellular Proteins (integral intensity 1702–1480 cm^−1^), Lipids (integral intensity 2988–2830 cm^−1^) and Nucleic Acids (integral intensity 1270–1190 cm^−1^) at different experimental steps for Cell1. The optical image of the cell acquired after Step 4 is also shown. Scale bar: Proteins (Min: 0 – Max: 10.3 a.u.); Lipids (Min: 0 – Max: 2.3 a.u.); Nucleic Acids (Min: 0 – Max: 0.65 a.u.). Relative intensity variations can also be deduced (0-1).

AFM analysis reveals that HEK293T formalin-fixed air dried cells have an epithelial morphology, i.e. polygonal shape with several pseudopodia (See Fig. 1a,f,k for Cell1, Cell2 and Cell3 respectively). Cells resemble a flattened pyramid, with a height profile that smoothly increases from the peripheral-cytoplasmic to the apical-nuclear compartment. Minimal differences encompassing local height variations at tens of nanometers scale or even less have been observed upon 1h30 dehydration under vacuum at Step1, while more pronounced changes from Steps 2 to 4 can be appreciated from Fig. 1. We could not identify significant lateral cellular shrinkage phenomena, often reported as a consequence of X-ray exposure; instead, an effect that becomes more evident with increasing dose is the degradation/thinning of the pseudopodia terminations. However, the most striking characteristic associated to X-ray irradiation is the increased inhomogeneity of the cell topography, where nanometric pits and bulges increase in number and size when increasing the dose. From the optical image in Fig. 2, which shows Cell1 at the end of the experiment, black spots corresponding to the AFM bulges can be seen. The collapse of cellular organelles and wrinkling of the cellular body have been reported by several authors as a consequence of the high tension forces undergone by the cell during air-dehydration^7^. Our results suggest these effects can be further enhanced by X-ray exposure in vacuum, a condition that encompasses both extensive dehydration and molecular fragmentation, as shown by SR-FTIRM results. Indeed, the most remarkable results are observed by this technique. In Fig. 2, chemical maps showing the distribution of cellular proteins, lipids and nucleic acids, related to each stage of the experimental design are plotted for Cell1 with a lateral resolution of 6 × 6 μm^2^ (Results for the other two cells are reported in the Supplementary Information). As a consequence of the 1h30 dehydration under vacuum, modest variations in the chemical plots could be noticed. Since cell thickness is barely affected by vacuum-drying when analysed with AFM (See Fig. 1a,b), small differences observed between the chemical profiles of the air-dried and vacuum-dried HEK293T cells are only due to a micrometric misalignment between sequential acquisitions. The intensity changes of the selected spectral bands for the unexposed air-dried cells can be up to 80% larger (see Fig. 2a), and reflects the pyramidal-like cellular profile, according to the Beer–Lambert law. Figure S6 in SI shows AFM images of the three cells at Step 0, resized accordingly to the lateral resolution of the chemical images (6 × 6 μm^2^ pixel size). The potential effects on the IR images induced by a misalignment of 3 microns in both x and y directions are simulated. This misalignment, that is a realistic estimation of the maximum error due to manual repositioning, can induce height variations in the order of 5–10% on each individual pixel, that would imply a comparable intensity variation of absorbance bands, assuming compositional invariance. Chemical profile differences between Step 0 and Step 1 are of the same order of magnitude, and indeed much lower than the ones revealed at the following exposure steps. As a matter of fact, from Fig. 2, it is possible to appreciate an outstanding decline of spectral intensity for all the aforementioned macromolecular cellular constituents upon X-ray exposure. Moreover, the extent of these variations clearly appears to be dependent on the cumulative dose: for the highest dose, the vibrational signals vanish almost completely. Establishing a semi-quantitative correlation between those changes and the concentration of fundamental cellular macromolecules would imply the normalization of the IR chemical maps to the cellular thickness. However, any attempt to perform this operation would produce unreliable results, due to a substantial difference on the lateral resolution provided by each technique. Nevertheless, relevant qualitative insights on the effects of X-ray exposure can be drawn by combining the two approaches. Once exposed to low doses, a significant decrease of the IR spectral intensities was observed, having a considerable decrement for Lipids (~20%) and still more pronounced for nuclear Proteins and Nucleic Acids (~25–30%). For medium exposure doses, the nuclear Nucleic Acids signal is almost suppressed, the Protein signal is approximately halved while the signal of nuclear Lipids is diminished ~30%. After LEXRF mapping, the signal of the nuclear Lipids is also suppressed, while solely the Protein signal can be detected. This trend is common to the other mapped cells (See Figures S1 and S3). The height variation obtained by AFM images of Cell1, averaged on the five experimental steps, has a maximum standard deviation of about 120 nanometers (~10% of the initial height) on a 6 × 6 pixel resized images (see Fig. S7 in SI). Therefore, AFM images do not highlight changes in cell volumes large enough to justify the dramatic effects pointed out by FTIRM. As observed, the order of magnitude of cell volume and absorbance variations is not comparable, implying a lack of correlation. Indeed, AFM shows rather punctual variations that encompass the formations of both nanometric holes and clumps, the effect of which is averaged in the micrometer range. In summary, the effects evidenced by FTIRM can be safely attributed to the destruction of the cellular vibrational network, the severity of which depends on the exposure dose.

**Figure 3:**
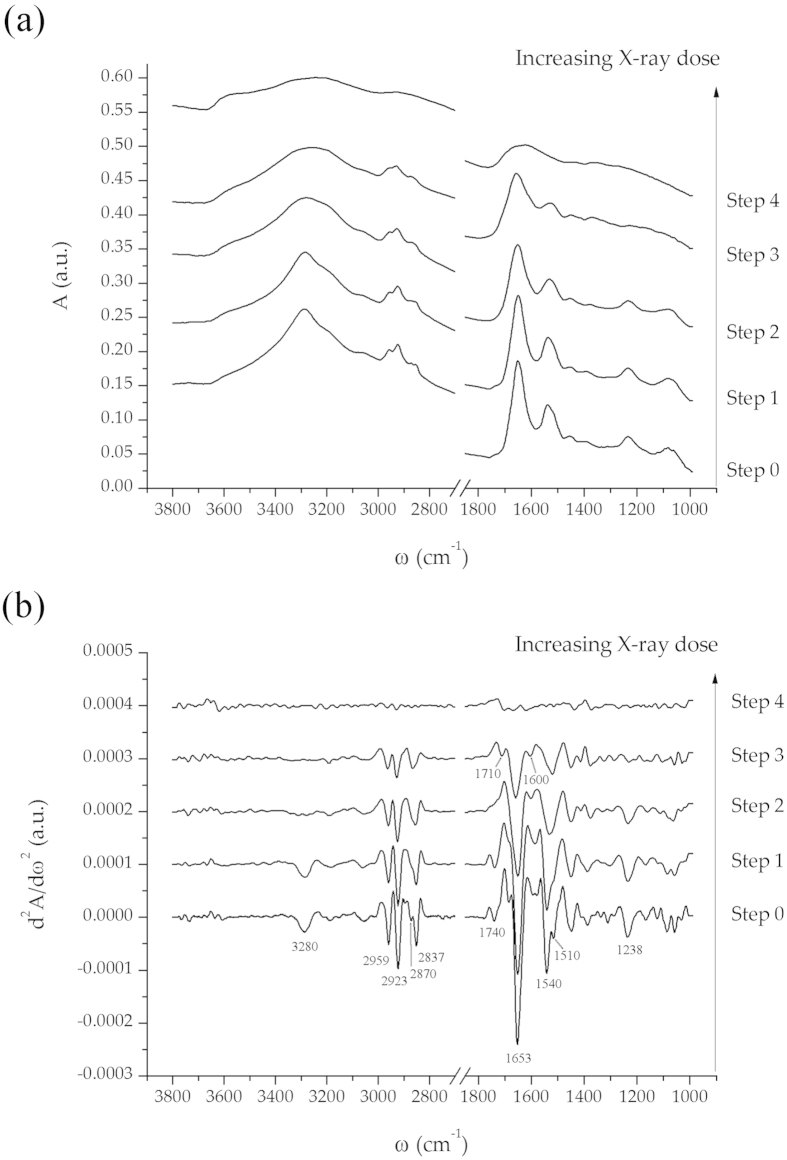
Absorbance spectra of the same nuclear point of HEK293T Cell1. **a**- Absorbance spectra of the same nuclear point of HEK293T Cell1 at the different experimental stages. Step 0 – air dried cells; Step 1 – vacuum dried cells; Step 2 – low dose exposure (2∙10^6^ Gray); Step 3 – medium dose exposure (2.2∙10^7^ Gray cumulative dose); Step 4 - High dose exposure (6.22∙10^8^ Gray cumulative dose). Each spectrum is presented with a 0.1 a.u. offset for clarity reasons. **b**- Second derivative spectra (Savitzky-Golay filter with 13 smoothing points) of the same nuclear point of HEK293T Cell1 at the different experimental stages. Each spectrum is presented with a 0.0001 a.u. offset.

More details on the occurring phenomena can be drawn from spectral analysis. To this aim, the same individual nuclear point in Cell1 has been selected (within the repositioning error) and the dose-dependent evolution of the absorbance spectrum and its derivative are plotted in Fig. 3a,b respectively. Air-dried and vacuum-dried cells are almost indistinguishable from a spectroscopic point of view. There are two prominent bands in the 1700–1480 cm^−1^ spectral region, Amide I and Amide II, related to vibrational modes of peptide backbone of cellular proteins (see Methods section for more details). The position of both bands is sensitive to the hydrogen bonding network of proteins, and therefore, to their secondary structure^8^. Despite that the complex matrix of the cellular environment hinders the possibility to disclose the finer structural details, noticeable information can still be drawn^0^. Specifically, the Amide I band is centered at 1653 cm^−1^ at both steps 0 and 1, revealing a prevalent α-helix conformation of cellular proteins^1^. Amide I maximum remains unaffected upon low-dose exposure, while an upshift to 1660 cm^−1^ is detected at Step3. The Amide II band, that has two contributions centered at 1540 cm^−1^ (α -helix protein domains^2^) and 1510 cm^−1^ (β-folded structures^3^) before X-ray exposure, is unbalanced toward lower wavenumbers at low and medium doses. However, the observed changes in spectral shape, driven by variations in the structural balance of cellular proteins^4^, seem to be modest in comparison to the intensity reduction. It should be stressed that these are formalin-fixed cells and that surely chosen fixation procedures affect the radiation damage experienced by the cells, as previously pointed out by other authors^6^. Formaldehyde is a non-coagulant cross-fixing chemical agent that acts by bridging via methylene (-CH_2_-) moieties primary and secondary amine groups of proteins^5^, mostly the NH_2_ end-group of lysine amino acid and amide group of peptide bond. The cross-linking reaction involves only close binding sites, thus allowing to preserve the secondary structure of proteins^6^. On this light, X-ray exposure implies a direct effect on the peptide backbone fragmentation, as also proven by the vanishing of the Amide A band contribution (NH stretching of peptide linkage) centered at ~3280 cm^−1^ and by the broadening of both Amide I and II bands, while intact protein domains seem to preserve their secondary structure almost unchanged up to the Step 3.

Similar considerations can be drawn for cellular lipids. The symmetric and asymmetric stretching bands of aliphatic chains do not shift until Step 3: methyl asymmetric and symmetric stretching bands are peaked at 2959 and 2870 cm^−1^ respectively, while the corresponding methylene bands are peaked at 2923 and 2847 cm^−1^. In addition, the intensity variation is more contained with respect to cellular proteins. However, the carbonyl ester band of phospholipids centered at ~1740 cm^−1^ 7 is not anymore detectable after low-dose exposure, revealing the enhanced propensity of the breakage of this linkage with respect to aliphatic C-H. Regarding nucleic acids, there is no trace of the phosphate backbone of DNA and RNA already at Step 3 (asymmetric stretching band of phosphate moieties centered at ~1238 cm^−1^), confirming the well-known susceptibility of the sugar phosphate backbone and DNA base-pairing to ionizing radiation^8^. It is however noticeable that the spectral features of the nitrogenous bases centered at 1600 (adenine and cytosine vibrations) and 1710 cm^−1^ (thymine and guanine vibrations)^9^ emerge from the background signal for both low and medium exposure doses.

As already pointed out, at the highest exposure dose, any vibrational detail is lost: the second derivative of the spectrum at Step 4 is dominated by spectral noise, while the absorbance spectrum by unnaturally broad bands. Such kind of spectra can be interpreted as a result from a quite heterogeneous and unordered mixture of IR active species. As a consequence of X-ray exposure to doses compatible with low energy XRF-mapping (LEXRF), cellular lipids, proteins, carbohydrates and nucleic acids lose their structure and both backbone and lateral chains of these macromolecules undergo an extensive fragmentation. Actually, at Step 4 Cell1 behaves like a dielectric sphere having a size comparable with the probing radiation, accordingly to Mie theory^0^. This further supports the hypothesis that very few vibrating moieties are intact upon the extensive soft X-ray exposure. The very same considerations can be drawn for Cell2 and Cell3 (See Figures S2 and S4).

**Figure 4:**
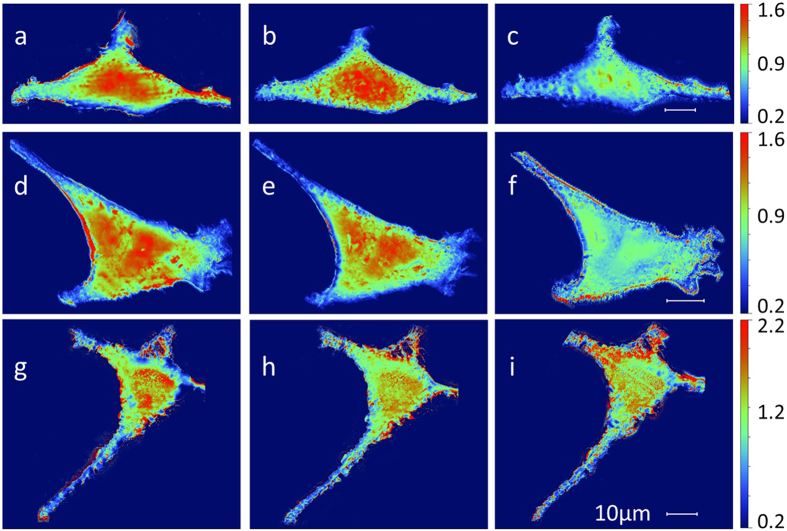
Density maps for three analysed cells. Density maps of the three analysed cells measured after the three different levels of soft X-ray doses: 2 × 10^6^ Gray (**a**), 2.2 × 10^7^ Gray (**b**) and 6.22 × 10^8^ Gray (**c**). The unit of measure on the scale bar is g/cm^3^.

Overall, FTIRM results suggest that the ionizing effects of low-energy X-rays on formalin fixed cells primarily induce the oligomerization of bio-macromolecules and then affect their constitutive monomers down to the formation of small, and possibly volatile, compounds, resulting in the comprehensive disintegration of the vibrational architecture. Cellular water residual upon air-drying could be one of those compounds, but decarbonylation and deamination reaction products could contribute as well^5^. Due to the complexity of the matrix and the specificity of the degradation mechanisms depending on sample type, preparation and type/dose of radiation, we cannot make further conjectures. However, the hypothesis of the formation of volatile compounds is supported by the density analysis obtained by combining X-ray absorption and AFM images. The density maps, obtained from AFM and absorption images, show indeed a progressive reduction of the cell average density with the increasing dose (Fig. 4). The results reported in literature are usually based on the evaluation of the mass thickness loss calculated from the corresponding absorption images^6^. The mass thickness can be calculated as follows:
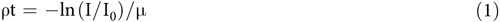
where ρ is the specimen density, t is the thickness, μ is the mass absorption coefficient of the cell, I the recorded transmission intensity at any particular point and I_0_ is the direct beam intensity (through the blank Si_3_N_4_).

On the other hand, in this work, we suggest a different approach where we explicitly show the density maps (ρ value point by point in the image), since we have measured the cell thickness t point by point using AFM microscopy. We believe that this approach is more comprehensive since it takes into consideration possible changes in cell volume as well. In any case, in order to be comparable with the literature, in the Supplementary Information we also show the trend of the mass thickness maps for each cell at each step of irradiation (Figure S3). As expected, the mass thickness decreases with increasing dose and this is already an indication of radiation damage consistent with previous studies. Nevertheless, by considering possible volume changes, our method provides more information on radiation effects. For instance, it could have been useful for Williams and co-authors^1^ to determine whether the observed chromosome shrinkage was present not only in the lateral dimension but also along the beam direction (perpendicularly to the sample support) and to which extent.

## Conclusions

To our knowledge, this is the first systematic study on the dose-dependent cellular radiation damage induced by STXM and LEXRF achieved by SR-FTIRM and supported by AFM. The AFM data analysis reveals a negligible variation in cell thickness as a function of vacuum drying and exposure, while X-ray absorption images, normalized over the cell volume, highlight a density variation that could not be ascribed to cell lateral shrinkage and increased cell-surface wrinkling (see Fig. 1). FTIRM unveils an overall reduction of the sample absorption as a function of the exposure dose, with an almost-complete loss of vibrational features for the highest dose (see Figs. 2,3). Our results confirm the detrimental effects of soft X-rays on the cellular architecture through the ionization of biomolecules and suggest possible evaporation of volatile compounds, generated as side products of the fragmentation events. Our findings are the results of soft X-ray exposure through STXM and/or LEXRF but similar observations apply for NEXAFS or XANES measurements with comparable doses and specimen preparation protocols.

The results presented in this paper provide new information related to the effect of X-ray exposure on formalin fixed cells. Beside mass thickness variations, previously assessed by other authors, here we demonstrate the potential of FTIR and AFM microscopies to determine radiation damage from biochemical, cell volume and cell density points of view. It must be noted that the non-destructive nature of these techniques does not induce any further damage while investigating the specimens. AFM analysis suggests the collapse of the cellular structure, enhanced by the covalent network disgregation highlighted by FTIRM. Therefore, special attention needs to be devoted to the interpretation of HR-STXM images and XRF maps, that could reveal unnatural ultrastructural details and/or elemental distributions induced by the probing source.

Similar observations could be of interest for samples coming from cultural heritage (such as polymer films, varnishes and dyes)^2^ or environmental science fields (such as soil and plants)^8^. Nevertheless, as mentioned in the introduction, due to the complexity of the radiation damage process, a careful evaluation should be done case by case, since the overall sample composition plays an important role on the preferential degradation pathways.

In conclusion, our study demonstrates that the combination of the three microscopy techniques provides an innovative approach and a step forward in understanding the effects of soft X-ray exposure, adding new information to what it is already known.

## Methods

### Sample preparation

Human Embryonic Kidney 293 cells (HEK293T) were cultured on silicon nitride membranes (100 nm thick) at 37 °C and 5% CO_2_ atmosphere in Dulbecco’s Modified Eagle Medium (DMEM) supplemented with 10% fetal Bovine Serum (FBS). Cells were left to attach overnight, and then fixed in 3.7% paraformaldehyde in Phosphate Buffer Solution (PBS) for 20 min at RT. Cells were then washed twice in physiological solution and finally quickly rinsed in DI water.

### Experimental Procedure

The steps followed for the accomplishment of the experimental design are summarized in Table 1.

For exposure to low and medium X-ray doses, cells were scanned in STXM mode at the TwinMic beamline of Elettra^3^ where a monochromatic sub-micron microprobe is delivered by a zone plate diffractive optic on the raster-scanned samples. The transmitted X-rays are collected point by point in the raster scan by a FRCCD camera^5^ allowing to construct absorption and phase contrast images, while the emitted X-ray fluorescence photons are acquired by 8 Silicon Drift Detector symmetrically facing the specimen^7^. In order to present a significant relevant case, excitation energy of 1 keV and a spatial resolution of 800 nm were chosen, representing typical experimental conditions for life science applications at TwinMic. In particular, the first two doses of 2∙10^6^ Gy and 2∙10^7^ Gy (cumulative dose 2.2∙10^7^ Gy) were delivered by scanning the cells in STXM with 20 ms and 200 ms acquisition time per pixel respectively. The highest dose of 6∙10^8^ Gy (cumulative dose 6.22∙10^8^ Gy) was provided during a typical TwinMic XRF mapping, where a 5 seconds acquisition time per pixel is required in order to obtain a detectable and meaningful XRF signal^7^. The dose was estimated considering the measurement parameters (incident flux, exposure time, geometrical parameters, excitation energy etc) as explained by Fayard and coworkers^0^. Specifically the imaging dose mainly depends on the scanning parameters (dwell time per pixel and pixel size), on the energy (1 keV) and on the cell composition (that affects the average absorption coefficient at the incident operation energy). The average cell composition was estimated as suggested from literature^9^. The incident beam intensity was measured by means of a photodiode placed directly downstream the focused spot.

FTIRM experiments were carried out at the SISSI beamline^0^ in Elettra – Sincrotrone Trieste. The life science branch of the beamline is equipped with a Bruker 70 interferometer coupled with the Hyperion 3000 Vis-IR microscope. Selected cells have been mapped with a lateral resolution of 6 μm, averaging 1024 scans per map point (scanner velocity 120 KHz, MCT-A detector 100 μm sensitive element).

AFM measurements were preformed using a JPK Nanowizard II coupled with a Zeiss Axiovert 200 inverted microscope. The images were acquired in intermitted contact mode, to minimize tip-to-sample interaction and sample deformations. The used tips are NanoWorld Arrow-NCR-50 while the cantilevers are 160 μm long, with a force constant of 42 N/m and a resonance frequency of 285 kHz. The oscillation amplitude was minimized to prevent alteration of the cells thus ensuring image quality and resolution.

### Data analysis

FTIR maps of measured HEK293T have been assembled with OPUS 7.0 software (Bruker Optics Ettlingen, Germany). Chemical images have been obtained using the same software, computing the integral of three distinct spectral regions. The area integral 2988–2830 cm^−1^ is representative of cellular lipids (Lipids); in this spectral region, symmetric and asymmetric stretching bands of methyl and methylene groups of aliphatic chains fall, that mostly characterize the chemical structure of cellular lipids. The area integral 1702–1480 cm^−1^ is representative of cellular proteins (Proteins); this region is dominated by Amide I (mainly stretching vibration of C =O groups of peptide linkage) and Amide II (mainly in plane bending of N-H of peptide linkage) bands, diagnostic for concentration and state of folding of cellular proteins. The area integral 1270–1190 cm^−1^ is mostly diagnostic of cellular nucleic acids (Nucleic Acids); the region is dominated by the asymmetric stretching of phosphate moieties of the DNA/RNA backbone. Due to the poor transparency to IR light on silicon nitride supports below 1150 cm^−1^, the symmetric stretching band of the phosphodiester backbone of nucleic acids could not be assessed, as well as most of the diagnostic bands for cellular carbohydrates. Second derivatives of spectra from selected cellular regions have been calculated by applying the Savitzky-Golay filter with 13 smoothing points.

AFM topographic analysis was performed using JPK image processing software that allowed us to monitor possible changes in cell volume at the different stages.

The cell density was estimated by using the X-ray absorption maps and the AFM images collected on the single cells at the different stages. The AFM measurements allowed us to evaluate the local cell thickness and to estimate the cell absorption coefficient point by point, following the procedure proposed and explained by Malucelli and coworkers^9^. This required careful alignment of the absorption and AFM images in order to have a proper overlap. Since the two techniques provide images with different lateral resolution, approximations and interpolations had to be made leading to some edge effects in the images and calculations. However this does not affect the overall results since the most interesting and useful information for our study concerns the nuclear and perinuclear regions, where both X-ray microscopy and FTIR signals are easily detectable.

## Author Contributions

AG and LV designed and coordinated the study. AG performed the X-ray Microscopy measurements, LV and DEB the FTIR ones, DC and ML the AFM maps. GK analysed the STXM data combined with AFM; SK and PS prepared the samples. AG, LV, GK and MK interpreted the data and draw the conclusions. The manuscript was written through contributions of all authors. / All authors have given approval to the final version of the manuscript.

## Additional Information

**How to cite this article**: Gianoncelli, A. *et al.* Soft X-Ray Microscopy Radiation Damage On Fixed Cells Investigated With Synchrotron Radiation FTIR Microscopy. *Sci. Rep.* **5**, 10250; doi: 10.1038/srep10250 (2015).

## Supplementary Material

Supporting Information

## Figures and Tables

**Table 1:** Scheme of the experimental design.

**Step**	**Process**	**AFM**	**FTIRM**	**STXM**	**LEXRF**
0	Air-drying (overnight)	✓	✓		
1	Vacuum drying (p <5∙10^−5^ mbar, 1h30)	✓	✓		
2	Low-dose exposure (2∙10^6^ Gy)	✓	✓	✓	
3	Medium-dose exposure (2∙10^7^ Gy)	✓	✓	✓	
4	High-dose exposure (6∙10^8^ Gy)	✓	✓		✓

At each step of the experiment, the performed measurements are marked.
