# Supplementary material for: Soft X-Ray Microscopy Radiation Damage On Fixed Cells Investigated With Synchrotron Radiation FTIR Microscopy
Source: Sci Rep. 2015 May 14;5:10250. doi: 10.1038/srep10250 (PMC4431353; doi:10.1038/srep10250)
Supplement: Supporting Information [file srep10250-s1.pdf]

Soft X-Ray Microscopy Radiation Damage On Fixed Cells Investigated With Synchrotron Radiation FTIR Microscopy

A. Gianoncelli[a]†, L. Vaccari[a]†, G. Kourousias[a]†, D. Cassese[b], D.E. Bedolla[a,c], S. Kenig[a], P. Storici[a], M. Lazzarino[b] and M. Kiskinova[a]

[a] Elettra – Sincrotrone Trieste, 34149, Basovizza, Trieste, Italy.
[b] CNR-IOM Laboratorio Nazionale TASC, 34149, Basovizza, Trieste, Italy.
[c] Physics Department, University of Trieste, 34127 Trieste, Italy

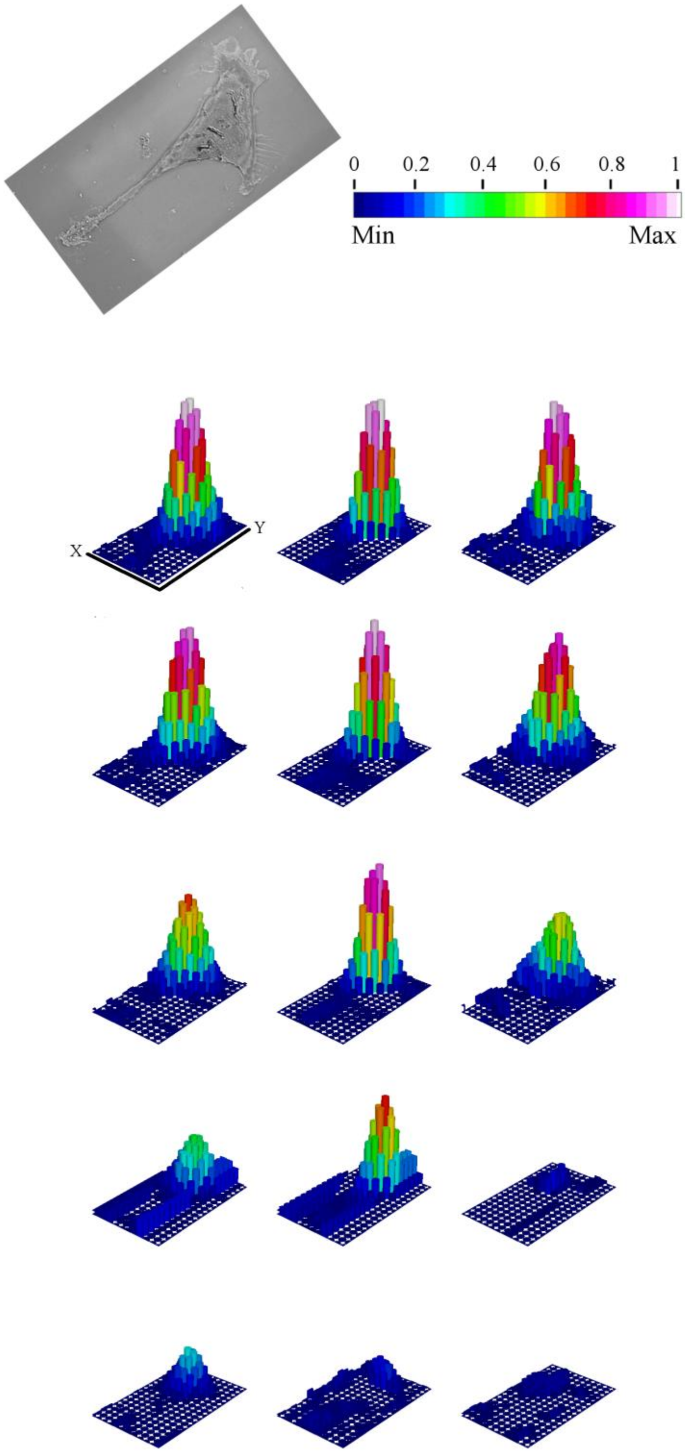

Figure S1: FTIR Chemical Images of Cell2. Chemical images (72X96 μm^2 area at 6 μm lateral resolution) of the distribution of cellular Proteins (integral intensity 1702-1480 cm^{-1}), Lipids (integral intensity 2988-2830 cm^{-1}) and Nucleic Acids (integral intensity 1270-1190 cm^{-1}) at different experimental stages for Cell2. The optical image of the cell was acquired after Step 4. Scale bar: Proteins (Min: 0 – Max: 9.7 a.u.); Lipids (Min: 0 – Max: 2.4 a.u.); Nucleic Acids (Min: 0 – Max: 0.64 a.u.). Relative intensity variations can also be deduced (0-1).

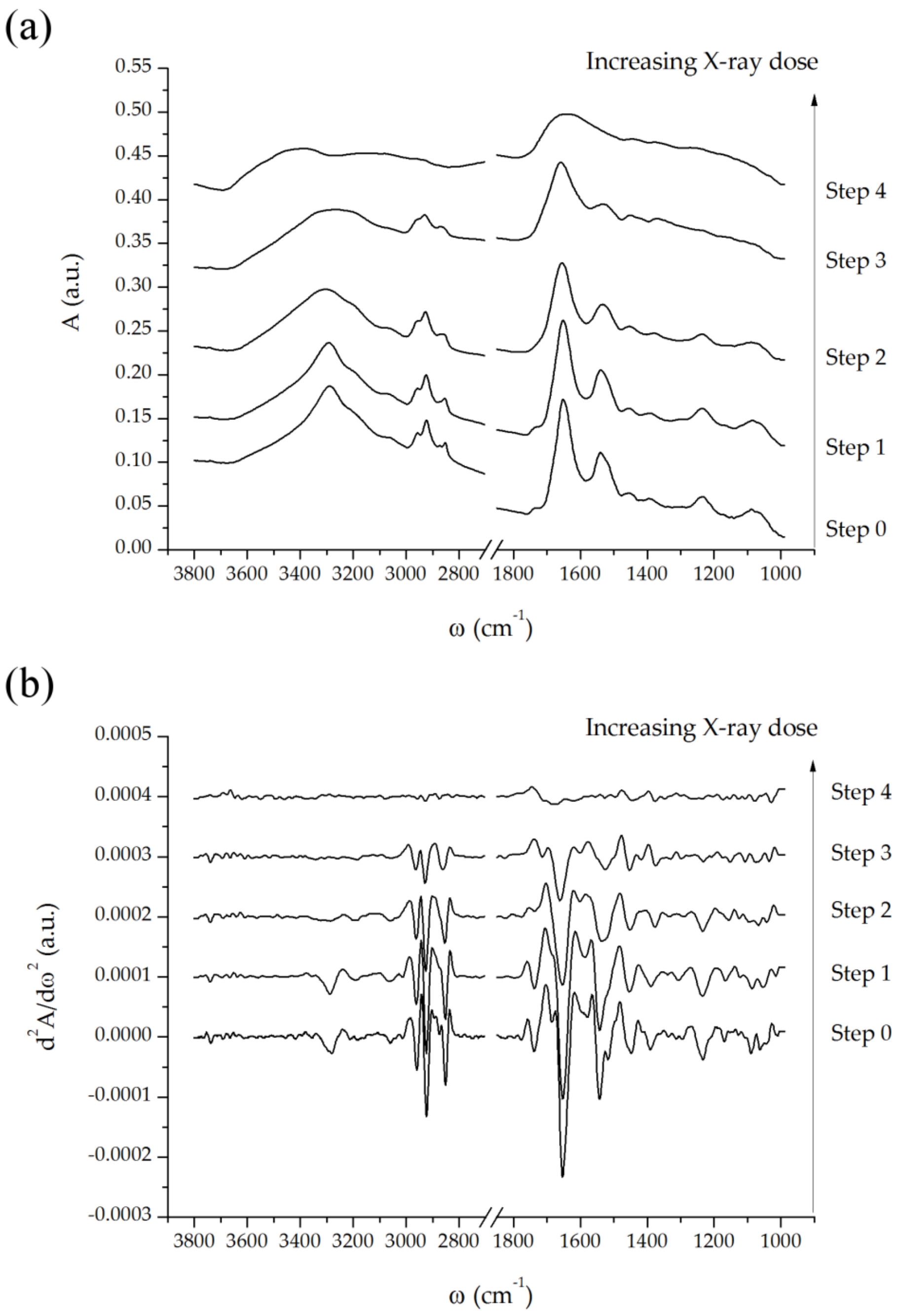

Figure S2: Absorbance spectra of the same nuclear point of HEK293T Cell2. a- Absorbance spectra of the same nuclear point of HEK293T Cell2 at the different experimental stages. Step 0 – air dried cells; Step 1 – vacuum dried cells; Step 2 – low dose exposure ($2\cdot 10^{6}$ Gray); Step 3 – medium dose exposure ($2.2\cdot 1^{07}$ Gray cumulative dose); Step 4 - High dose exposure ($6.22\cdot 10^{8}$ Gray cumulative dose). Each spectrum is presented with a 0.1 a.u. offset for clarity reasons. **b-** Second derivative spectra (Savitzky-Golay filter with 13 smoothing points) of the same nuclear point of HEK293T Cell2 at the different experimental stages. Each spectrum is presented with a 0.0001 a.u. offset .

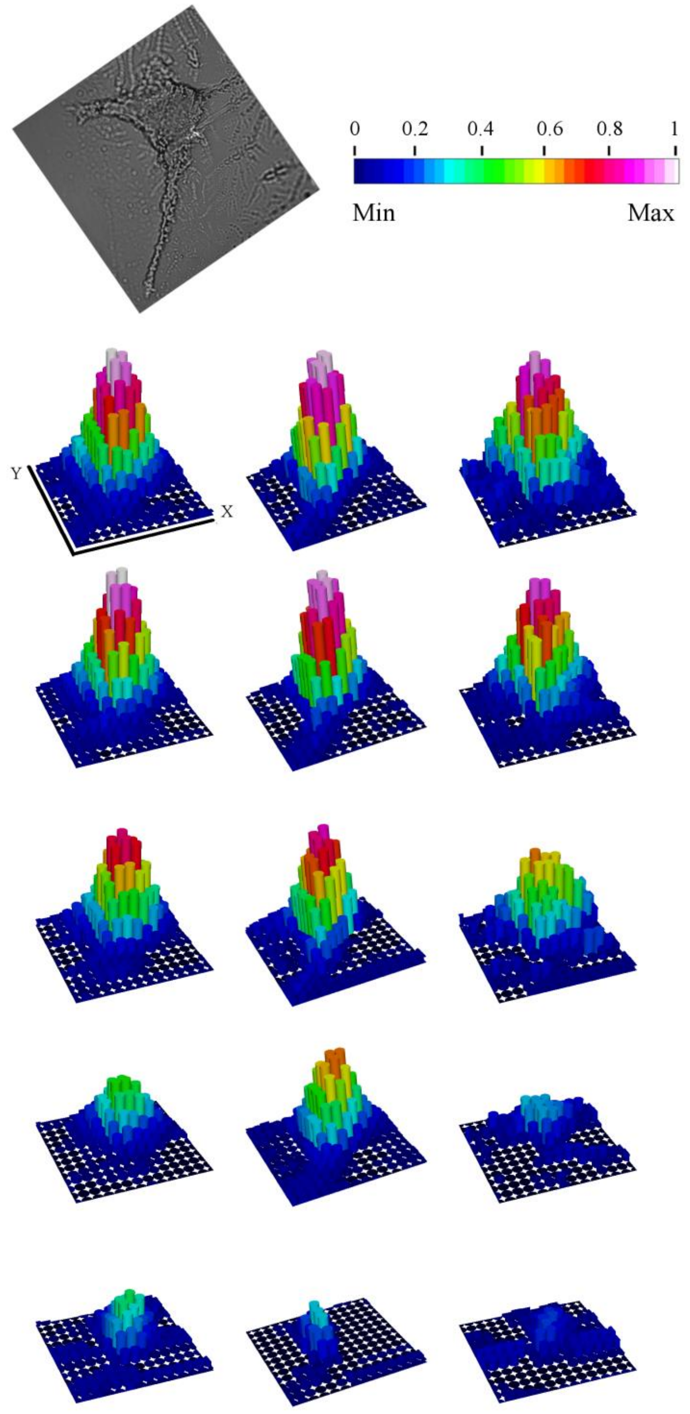

Figure S3: FTIR Chemical Images of Cell3. Chemical images (84X84 μm^2 area at 6 μm lateral resolution) of the distribution of cellular Proteins (integral intensity 1702-1480 cm^{-1}), Lipids (integral intensity 2988-2830 cm^{-1}) and Nucleic Acids (integral intensity 1270-1190 cm^{-1}) at different experimental stages for Cell3. The optical image of the cell was acquired after Step 4. Scale bar: Proteins (Min: 0 – Max: 9.4 a.u.); Lipids (Min: 0 – Max: 1.75 a.u.); Nucleic Acids (Min: 0 – Max: 0.6 a.u.). Relative intensity variations can also be deduced (0-1).

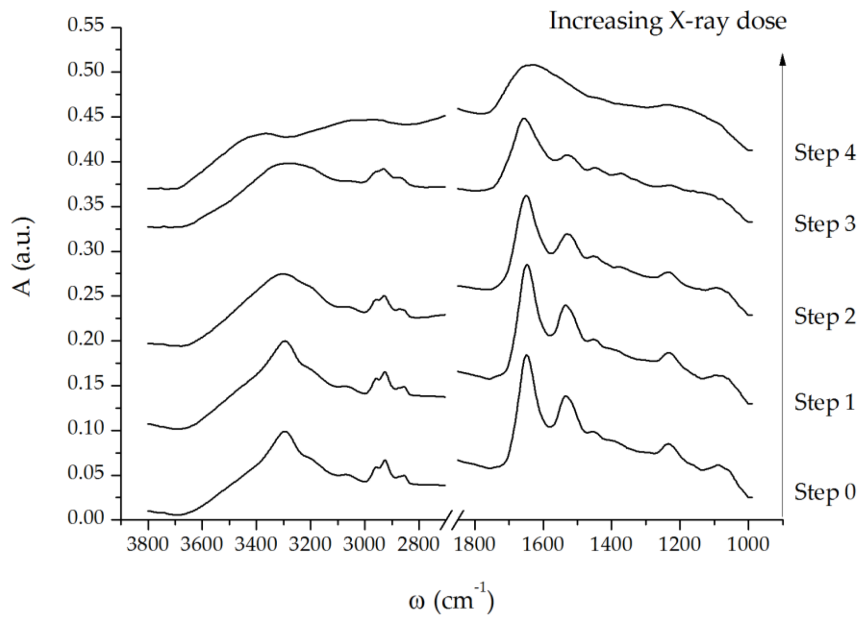

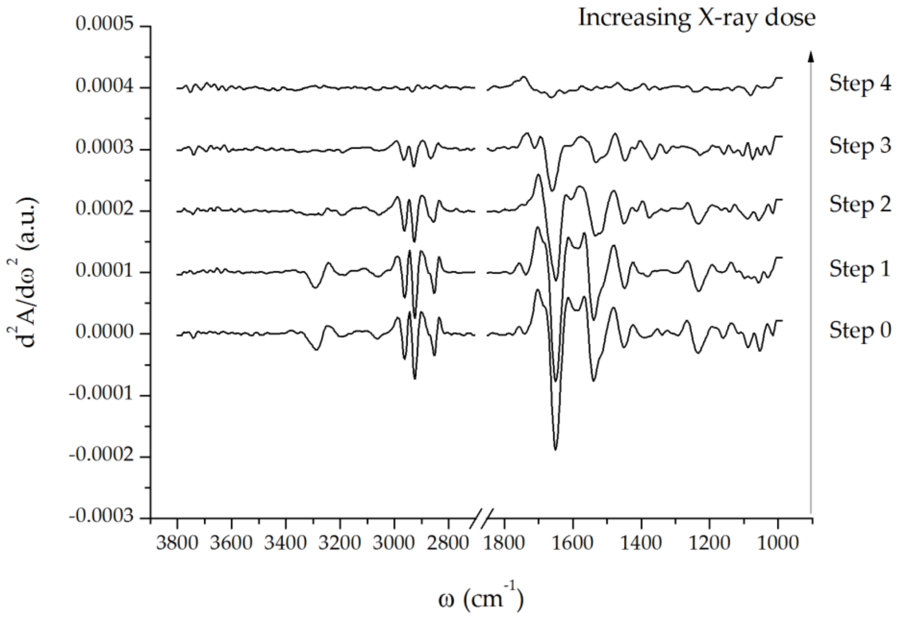

Figure S4: Absorbance spectra of the same nuclear point of HEK293T Cell3. a- Absorbance spectra of the same nuclear point of HEK293T Cell3 at the different experimental stages. Step 0 – air dried cells; Step 1 – vacuum dried cells; Step 2 – low dose exposure ($2{\cdot}10^{6}$ Gray); Step 3 – medium dose exposure ($2.2{\cdot}1^{07}$ Gray cumulative dose); Step 4 - High dose exposure ($6.22{\cdot}10^{8}$ Gray cumulative dose). Each spectrum is presented with a 0.1 a.u. offset for clarity reasons. **b-** Second derivative spectra (Savitzky-Golay filter with 13 smoothing points) of the same nuclear point of HEK293T Cell3 at the different experimental stages. Each spectrum is presented with a 0.0001 a.u. offset .

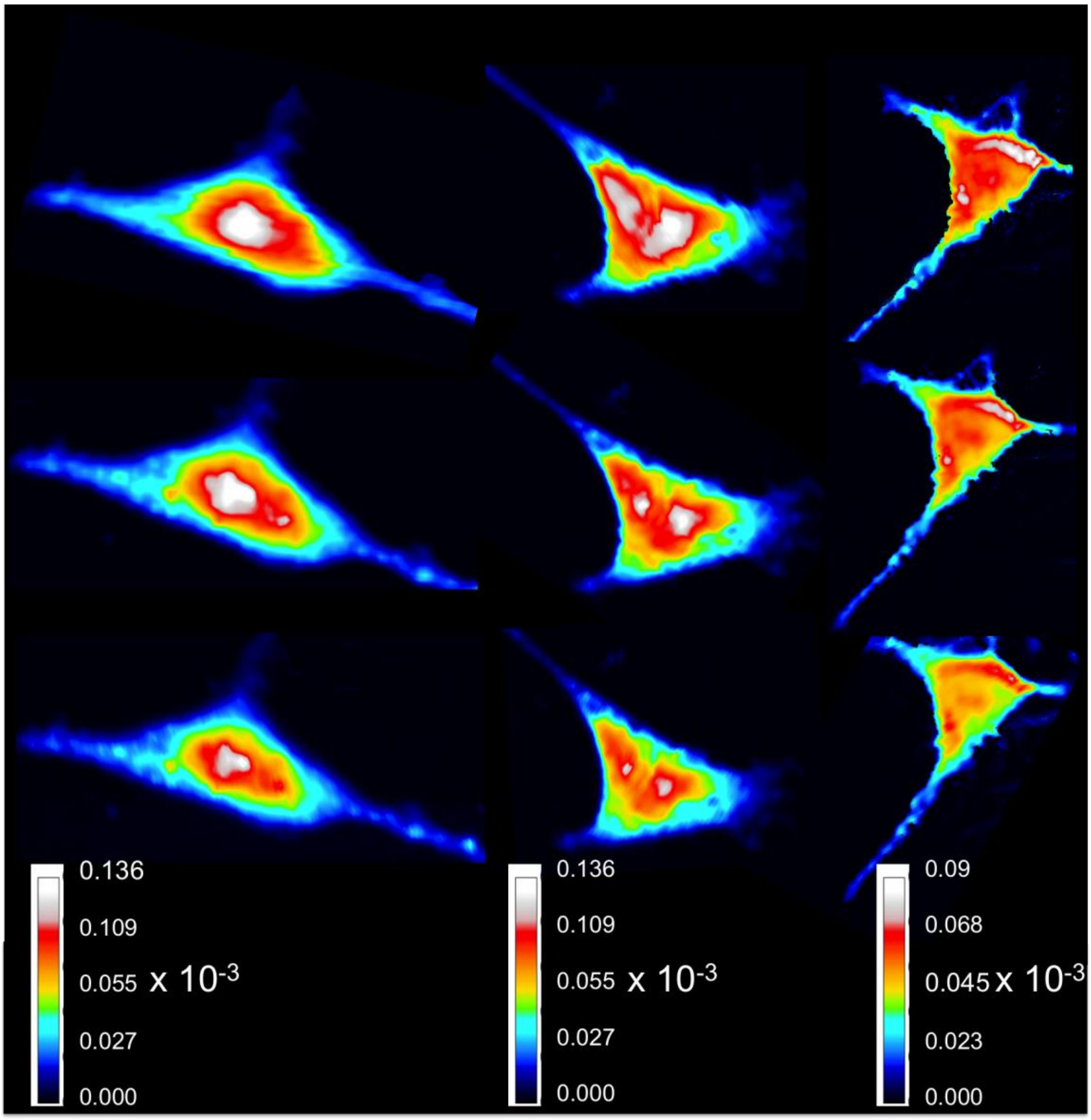

Figure S5: Mass thickness maps of the three analysed cells. Mass thickness maps calculated according to equation (1) for each analysed cell at the different X-ray doses: Step 2 (a, d, g), Step 3 (b, e, h) and Step 4 (c, f, j). The unit of measure on the scale bar is g/cm^2.

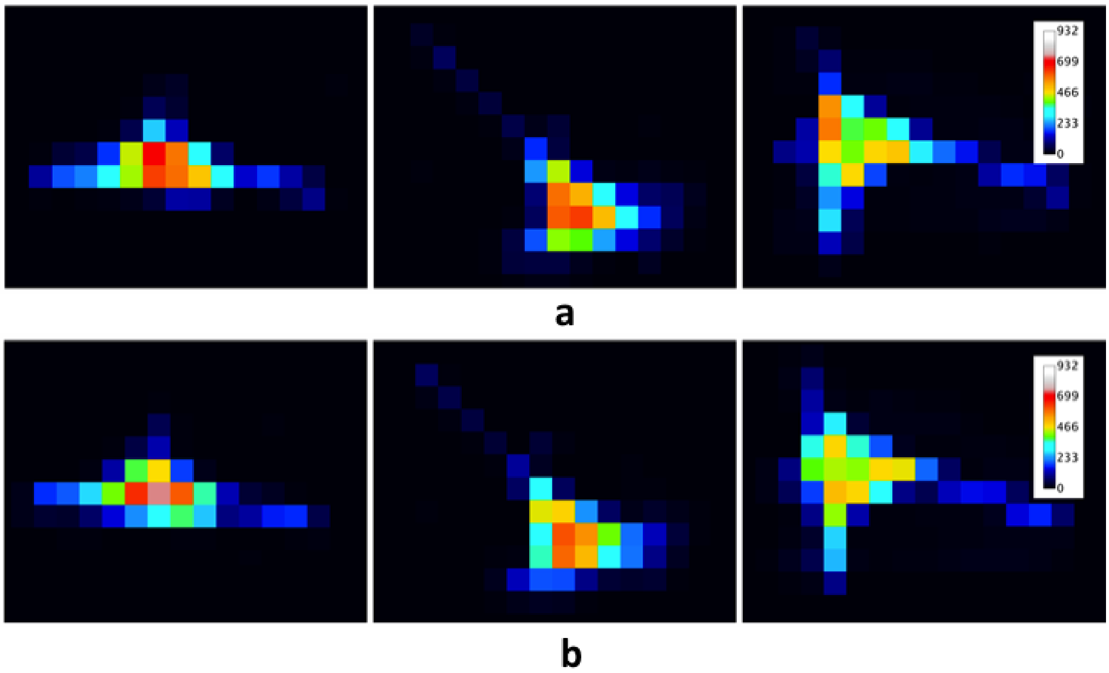

Figure S6: AFM images under-sampled at the spatial resolution used in FTIR spetromicroscopy. AFM images of the three cells at Step 0, resized accordingly to the lateral resolution of the chemical images (6x6 μm^2 pixel size). Images in Panel b are misaligned of 3 microns in both x and y directions compared to the plots of Panel a, simulating a realistic estimation of the maximum repositioning error.

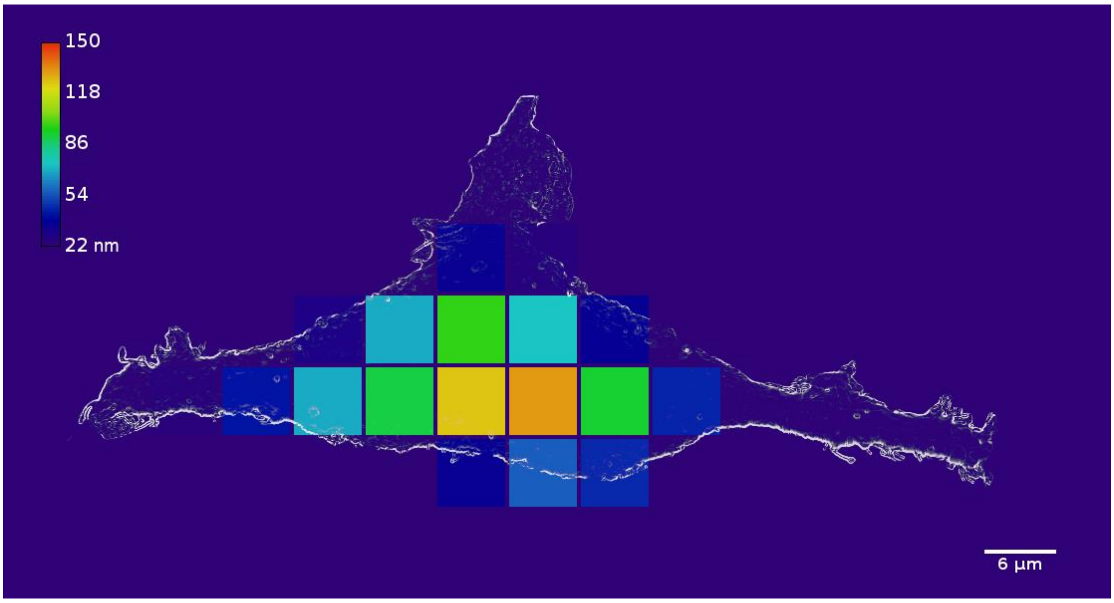

Figure S7: Standard deviation of the five AFM images of Cell1. Standard deviation of the height variation of Cell1 evaluated on the 5 steps, averaged over 6x6μm^2 areas corresponding to the spatial resolution of the FTIRM images.
